# Photonic crystal and quasi-crystals providing simultaneous light coupling and beam splitting within a low refractive-index slab waveguide

**DOI:** 10.1038/s41598-017-01842-w

**Published:** 2017-05-12

**Authors:** Jingxing Shi, Michael E. Pollard, Cesar A. Angeles, Ruiqi Chen, J. C. Gates, M. D. B. Charlton

**Affiliations:** 1 Faculty of Physical Sciences and Engineering, Building 53, University of Southampton, Southampton, UK; 2 School of Photovoltaic and Renewable Energy Engineering, Tyree Energy Technologies Building, UNSW Australia, Sydney, Australia

## Abstract

Coupling between free space components and slab waveguides is a common requirement for integrated optical devices, and is typically achieved by end-fire or grating coupling. Power splitting and distribution requires additional components. Usually grating couplers are used in combination with MMI/Y-splitters to do this task. In this paper, we present a photonic crystal device which performs both tasks simultaneously and is able to couple light at normal incidence and near normal incidence. Our approach is scalable to large channel counts with little impact on device footprint. We demonstrate in normal incidence coupling with multi-channel splitting for 785 nm light. Photonic crystals are etched into single mode low refractive index SiON film on both SiO_2_/Si and borosilicate glass substrate. Triangular lattices are shown to provide coupling to 6 beams with equal included angle (60°), while a quasi-crystal lattice with 12-fold rotational symmetry yields coupling to 12 beams with equal included angle (30°). We show how to optimize the lattice constant to achieve efficient phase matching between incident and coupled mode wave vectors, and how to adjust operating wavelength from visible to infrared wavelengths.

## Introduction

Couplers and power splitters are key components for integrated optics and have been studied extensively for decades^1^. Grating couplers are usually used to couple light into optical waveguides in order to overcome the mode mismatch issue between the waveguide mode and fibre mode. They also allow wafer scale test for devices on *SOI* substrates^2, 3^. There are many grating coupler designs with potential for high efficiency and good alignment tolerance for fibre-to-chip coupling^4, 5^. Once coupled, light is typically guided to the input of a beam splitter using etched waveguides^6–8^ thereby providing splitting of optical signals. Common splitter implementations include multimode interference filters (MMI)^9, 10^ or Y-junction splitters^11, 12^. Using a Y-junction splitter approach, the overall size of the combined system becomes very large (of the order of mm) for large channel counts (>8) ^13^. MMI splitters have the advantages of near equal power output over the output channels, and small overall footprint size. However, the optical power throughput of MMI splitters is low due to the requirement for single mode input/output waveguides. These two approaches also require rib or ridge waveguide structures to be etched, incurring loss due to waveguide side wall roughness^14^. The need for waveguide tapers (or integrated reflectors) usually limits the input light source to a single mode fibre positioned close to the surface with few micron coupling spot area. Silicon Photonics applications generally only need single mode input coupling due to the nature of the applications. Many applications such as Bio-sensing do not require fibre coupling, and can benefit from larger area free space coupling from light sources such as LEDs and Laser devices. They can benefit from higher power input coupling from highly multimode light sources. Hence our device targets different application areas than conventional grating coupler devices.

Photonic crystal based power splitters have recently been demonstrated for ‘in-plane’ waveguide configurations, but the number of power-split channels was limited to two, and were also polarization dependent^15–17^. They also did not provide dual functionality. To date, there have been few attempts to perform free space light coupling and power splitting function in one single device in order to decrease the size, and cost, of the full chip.

Most of these approaches were also based on high index contrast waveguides such as SOI working at IR wavelengths^18, 19^. Silicon is particularly common due to its importance in integrated optics and compatibility with CMOS devices. However, low refractive index materials are also highly useful in the fields of biological and chemical optical sensing^20^.

Dual functionality in a single device is often preferred for integrated optical devices^21–23^. In this paper, we report the design and fabrication of couplers performing the dual functionality of coupling and splitting functions for 785 nm light. These are based on two-dimensional photonic crystal (PC) and photonic quasi-crystal (PQC) lattices with the benefit of very small footprint (microns rather than mm for the combined system). These devices are incorporated into low refractive silicon oxynitride (SiON) waveguides, which despite being a low refractive index material, is still CMOS compatible. This material (SiON) can also be deposited onto glass substrates enabling rear side light coupling which is useful for large area sensing applications. The lattice points are formed by etched holes. The wavelength of 785 nm was chosen for compatibility with sensing applications^24–26^. Couplers based on periodic and quasi periodic 2-dimensional photonic crystal lattices provide several key advantages. Firstly, coupling over a large azimuthal angular range with a particular incidence angle (especially normal incidence) is possible. Secondly the lattice can be tailored such that there is improved mode matching to the circularly symmetric Gaussian shape of the incident laser beam. The third and the most important factor is that they can simultaneously act as both couplers and beam splitters for the incident beam. The final point is that they provide very high optical power throughput since the in-plane split beams are laterally multi-moded, as would be the case for a very wide rib/ridge waveguide. We utilize this feature to yield multiple in-plane propagating modes in a slab waveguide. Furthermore, a functional waveguide taper component is not needed in this configuration, and the coupling area can be scaled in size to match the input source dimensions.

## Design of photonic crystal and Photonic Quasi-Crystals Coupler

**Figure 1:**
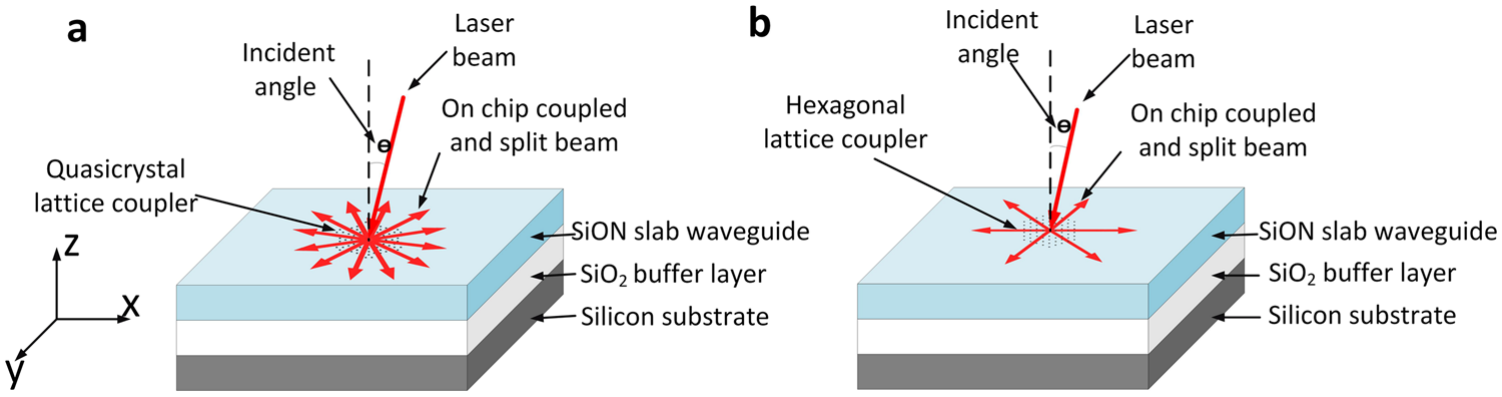
Schematics of light coupling and splitting on photonic crystal structures. (**a**) Quasi-crystal lattice and (**b**) triangular lattice couplers/splitters.

The schematic diagram is shown in Fig. 1. The 1 × 6 coupler is based on a triangular lattice photonic crystal and the 1 × 12 coupler is based on a quasi-crystal constructed by random Stampfli inflation^27^. All of the structures are etched into a 400 nm thick SiON slab waveguide deposited on top of a 2 µm SiO_2_ buffer layer on a silicon substrate. Variation of the coupling angle around the surface normal is explored via modelling and optimization using commercial software (RSoft DiffractMOD).

The coupling and power splitting principle is based on Bragg diffraction. When light hits the photonic crystal at near normal incident angle (θ) shown in Fig. 1, the incident light will be diffracted into several coupled modes. The maximum number of coupled modes depends on the rotational symmetry of the photonic crystal lattice, as defined by the number of primary Bragg spots in its diffraction pattern. At near normal incidence, splitting is symmetric with equal included in-plane (xy plane) angle. Lower order diffracted light, which matches the waveguide modes, is then confined in the slab waveguide.

Designing photonic crystal couplers for normal incidence coupling basically requires two major steps. Firstly, the diffraction pattern of the lattice is investigated to determine the location of the primary Bragg peaks. The diffraction pattern is essentially the reciprocal space (frequency domain) representation of the lattice. Secondly, to achieve phase matching, lattice parameters must be chosen such that the ‘in-plane’ component of the wave-vector corresponding to the photonic crystal Bloch mode, matches the location of the primary Bragg peaks. The photonic crystal Bloch mode wave-vector depends strongly on the slab waveguide material and thickness, and the photonic crystal fill factor.

**Figure 2:**
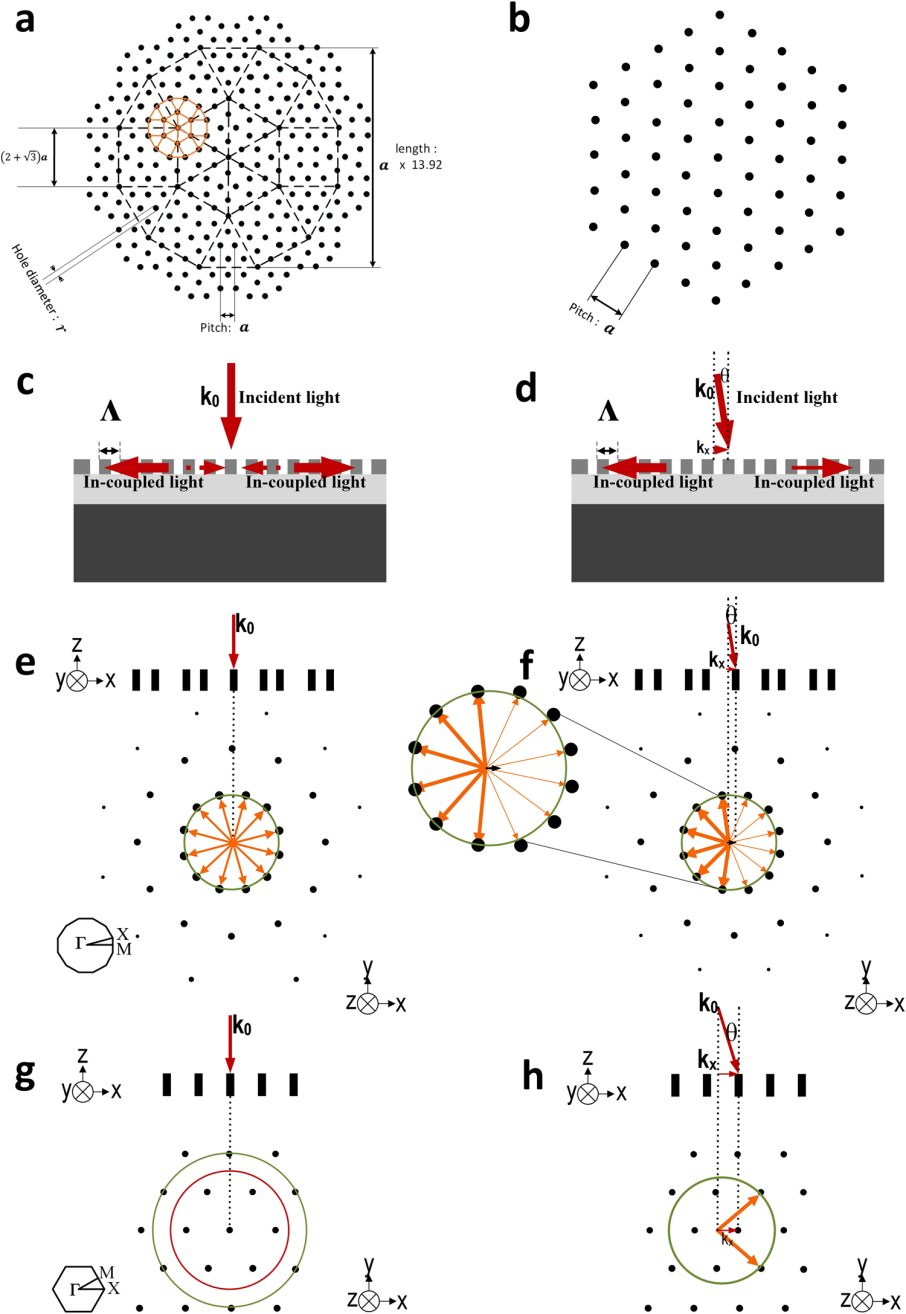
Comparison of triangular lattice and quasi-crystal lattice in free space and reciprocal space. (**a**) Real-space lattice of square-triangle tiling random-Stampfli quasi-crystal lattice structure. (**b**) Real-space lattice of the triangular lattice structure. (**c**) Schematic cross-section showing normal incidence coupling to the waveguide mode. (**d**) Schematic cross-section showing near-normal incidence coupling to the waveguide mode. (**e**) Ewald circle construction of a quasi-crystal lattice in reciprocal space with normal incidence coupling. (**f**) Ewald circle construction of a quasi-crystal lattice in reciprocal space with near-normal incidence coupling. (**g**) Ewald circle construction of a triangular lattice in reciprocal space showing the light-line in red. (**h**) Ewald circle construction of a triangular lattice in reciprocal space with a large offset-incidence angle. Green circles represent the Ewald circle construction. Yellow arrows indicate the allowed coupled mode propagation directions and the width of the arrows schematically indicate the strength of each scattered beam.

Conventional 2-dimensional photonic crystal lattice shapes such as square and triangular lattices have symmetries limited to four and six-fold respectively. It is not practical to efficiently achieve higher ratios of beam splitting by increasing the diameter of the Ewald circle, since the first order Bragg peaks are the strongest and also due to waveguide mode confinement conditions, relating to the ‘light line’ (conditions for total internal reflection, an indicative light line is shown as a red circle in Fig. 2g). If the diameter of the Ewald circle exceeds that of the ‘light line’ then light will consequently leak directly out of the guide and coupling will be very weak.

However, higher order symmetry lattices can be achieved by using quasi-crystal designs. For example octagonal, dodecagonal and decagonal quasi-periodic photonic crystals display 8, 10 and 12-fold rotational symmetry, respectively^28, 29^. According to the class of quasi-crystal lattice, and the associated mathematical construction algorithm, an arbitrary level of symmetry can be achieved^30^, and so consequently more Bragg peaks will be within the light line allowing higher number of split beams.

Unlike the periodic triangular lattice, a large number of cells (rods/holes) are required to construct the quasi-crystal structure in order to preserve the long term periodicity of the lattice. Here, a square-triangle tiling random-Stampfli inflation rule is used to produce a lattice exhibiting long-range 12-fold symmetry^31^. The dodecahedral cell is inflated by the Stampfli inflatio﻿n factor of $2+\sqrt{3}$ and acts as the parent cell (black dash lines in Fig. 2a) to generate offspring dodecagons. One example of offspring dodecagon is shown as yellow solid lines in Fig. 2a. The total size of the parent cell is **a** × 13.9, where **a** is the distance between lattice points (small black circle). Figure 2e shows the 12-fold quasi-crystal diffraction pattern and the diffraction strength is proportional to the Fourier coefficients^32^.

It is instructive to visualize the phase matching process using Ewald construction^33^. Although higher order Bragg peaks can be used for coupling, the primary Bragg peaks (corresponding to first order diffraction conditions) yield the strongest coupling efficiency^34, 35^ and typically eliminates additional out-of-plane diffraction losses.

The real-space lattice structure for the quasi-crystal and triangular photonic crystal structure are shown in Fig. 2a and b. The diffraction patterns (reciprocal lattice) are illustrated in Fig. 2e–h and in Figure S1. The diffraction patterns clearly demonstrate the expected 6-fold rotational and 12-fold rotational symmetry, with the strongest points (primary Bragg peaks) located in the inner circle (shown in green in Fig. 2). The 1st Brillouin zone and Γ point are shown as insets in Fig. 2e and g.

Normal incidence requires that the k vector of the incident light source is perpendicular to the surface of the 2D photonic crystal slab. An off-normal coupling angle is used in our experiment because the fabrication tolerance of these photonic crystal and quasi-crystal, in terms of hole size and shape, are not perfect to the design specifications, and this slightly affects the coupling conditions and beam splitting ratio.

According to the design specifications, exactly vertical incidence should achieve coupling and multi-beam splitting, whereas due to small imperfections in fabrication for our demonstrator device, coupling is achieved at a small offset angle. Figure 2 show the vertical coupling and near normal incident coupling concept and how the beam splitting will be affected according to the theory. Figure 2c and d shows the concept of in-coupled guided modes in photonic crystal/photonic quasi-crystal with normal incident light and near normal incident light (*θ*) respectively.

In Fig. 2c and d the solid red arrows indicate in-coupled light and the dotted red lines indicate unwanted reflections in the ‘normal incidence’ coupling scenario. These back-reflections can be avoided with ‘near normal incidence’ coupling. The comparison between ‘normal incidence’ coupling and ‘near normal incidence’ coupling is shown in Fig. 2e and f for quasi-crystal and Fig. 2g and h for triangular lattice photonic crystals. Figure 2e–h shows a reciprocal lattice space representation of the lattice pattern (Fourier transform), and we follow the Ewald construction to predict coupling conditions as follows:

We draw a vector with a length of *k*_*x*_ terminating at one of the reciprocal lattice points. *k*_*x*_ is equal to 0 for normal incidence angle and *k*_0_sin *θ* for an incidence angle of *θ* respectively, as indicated on the *x-z* plane cross section above Fig. 2e–h. *k*_0_ is the magnitude of the incident vector at the operating wavelength. The Ewald circle is superimposed as a green circle with a diameter matching the ‘in-plane’ mode propagation constant. This is centred at the reciprocal lattice ‘origin point’ *k*_0_. For multi-beam coupling to occur, it is necessary for the Ewald circle to intersect a number of reciprocal lattice points (in k-space). Under perfect conditions (Fig. 2c), the propagating wave-vectors are phase matched to the lattice, and so the yellow arrows in (Fig. 2e) indicates the allowed coupled mode propagation directions. The width of these arrows schematically indicates the strength of each scattered beam, in this perfect scenario the coupling strengths are equal.

If the Ewald construction interacts with higher order Bragg peaks, this may result in the in-plane modes leaking from the core layer. This is due to the light line (show as a red circle in Fig. 2g) being within the Ewald circle. The radius of the red circle corresponds to the maximum possible value of the modal propagation constant for the SiON slab waveguide.

If the incidence angle θ is slightly near-normal (as shown in Fig. 2d), then the centre of the Ewald circle will shift position, and no longer corresponds to the ‘origin reciprocal’ lattice point and results in the case shown in Fig. 2f. An inset shows a magnified view in Fig. 2f, highlighting the slight shift in the centre position and so intersects different first order Bragg peaks (reciprocal lattice points). This results in a variation in the coupled beam strengths and the number of split beams. A large offset-incidence angle will result in reduced beam splitting. In Fig. 2h we show this effect in relation to a triangular lattice. In this case only 2 lattice points intersect with the Ewald circle and it results in 2 split beams.

**Figure 3:**
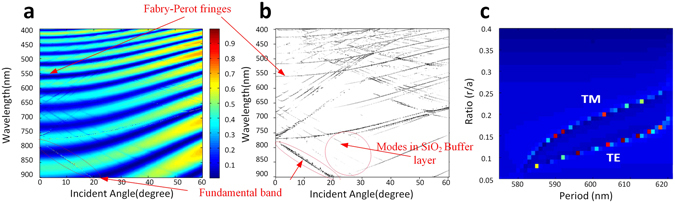
Angle-resolved zero-order reflectance map of a triangular lattice coupler showing the coupling angle is near 0 degree at 785 nm. (**a**) Raw simulation data. (**b**) Filtered simulation data with extracted bands. (**c**) Zero-order reflection at normal incidence and 785 nm as a function of different photonic crystal lattice period and hole radius-to-period ratio (r/a) for triangular photonic crystal lattice.

In order to predict the coupling angle, we need firstly to determine accurately the centre of the Ewald circle in the reciprocal lattice. As explained above, a small ‘Offset-Incidence’ angle will change the projected in-plane k-vector (*k*_*x*_), and will affect both the splitting beam directions and number. Optimal coupling angle for any given operating wavelength (785 nm in this case) can be obtained from the simulated angle-resolved dispersion map, shown in Fig. 3a and b. We then apply this to the Ewald construction method (described above) to determine the corresponding beam splitting conditions corresponding to the exact parameters of the photonic crystal/quasi-crystal lattice. From the Ewald circle construction, the required lattice constant and air hole diameter for the quasi-crystal and triangular structure were roughly found to be 570 nm/100 nm and 575 nm/114 nm, respectively.

We showed that the directions of diffracted beams for any given incident wave vector are determined by the Ewald construction. A prerequisite for building the Ewald circle is that the magnitude of the reciprocal lattice vectors correspond to the primary Bragg peaks. To achieve uniform symmetric coupling and splitting, it is necessary to couple incident light at normal or near normal incidence to the surface. This yields 6 split beams for the triangular coupler and 12 split beams for the 12-fold quasi-crystal coupler. The centre of the Ewald circle lies at the centre of the circle of primary Bragg peaks. The primary Bragg peaks are represented by the first order diffraction according to Bragg’s law. The condition for first-order diffraction is given by equation. (1):

1 $${\rm{\Lambda }}=\frac{\lambda }{{n}_{eff}-\,\sin \,\theta }$$

where Λ is the distance between two lattice points (black filled circle), *n*_*eff*_ is the effective index of the stable photonic crystal waveguide mode, *λ* is the free-space wavelength, and *θ* is the incident angle (*θ* is depicted in Fig. 1) relative to the surface normal. In order to construct the Ewald circle, we convert the equation above to its reciprocal space representation to give equation (2):

2 $$\beta -\,{k}_{0}\,\sin \,\theta =G$$

where *β* is the mode propagation constant (*k*_0_ n_*eff*_), *k*_0_ is the wave vector in free space and *G* is a reciprocal lattice vector given by 2*π*/*a*. The mode propagation constant (in-plane wave vector) in the photonic crystal area lies between *k*_0_ *n*_*core*_ and *k*_0_ *n*_*sub*_ and is lower than the effective index of the SiON slab waveguide mode. The lattice fill factor (*f* ) is introduced in order to calculate an approximate effective index for the photonic crystal mode. Fill factor is defined as the volume ratio between the air holes and the whole pattern area. The triangular structure gives the fill factor expression as equation (3):

3 $${f}_{triangular}=2\pi {(r/{\rm{\Lambda }})}^{2}/\sqrt{3}$$

where $r$ is the radius of the air holes. Using this fill factor, we can derive the estimated refractive index of the photonic crystal mode^36^ as equation (4):

4 $${n}_{eff}=(1-f){n}_{core}+f\times {n}_{air}$$

where *n*_*air*_ is the refractive index of air, which is normally equal to 1.

The last required parameter is the effective index of the modes supported by the slab waveguide. The refractive index of the SiON film was extracted experimentally by ellipsometry measurements of a SiON thin film and was found to be 1.7 at 785 nm. Mode solving (using Lumerical FDTD solutions software) reveals that the core thickness must be less than 600 nm for single mode operation. In this work a SiON thickness of 400 nm is used. Using these parameters the TE_00_ and TM_00_ mode indexes were calculated to be 1.5881 and 1.5560 respectively. However, it should be noted that the tendency for the lower order photonic crystal modes to be largely concentrated in the high index material skews these results.

Iterative approaches are require to more accurately determine the true value of *n*_*eff*_. According to the normal incident condition, the coupling angle *θ* should be 0° (Γ point). But there will be a tradeoff between diffraction efficiency and even splitting of the beams. Normal incidence to the photonic crystal surface results in even beam splitting to n-channels, however it also allows ‘in-plane’ second order Bragg reflection back into incident space^37^, reducing total coupled power.

Computer modelling of the triangular lattice coupler was performed using the Rigorous Coupled Wave Analysis method (RSoft DiffractMOD). The total reflection is calculated for a wide range of incident angles (*θ*) and wavelengths. The light source was launched from 0 degree to 60 degree and the wavelength range was from 400 nm to 900 nm. This produces the angle-resolved dispersion map of the triangular lattice shown in Fig. 3a. The dispersion bands are extracted from the original raw data via gradient analysis and a peak detection algorithm shown in Fig. 3b. This method is far preferable to conventional plane wave band structure calculations as the results reveal all information about the 3-dimentional system (including substrate and over-layers), and are directly comparable to experimental measurement as shown later on.

In the angle-resolved zero-order reflectance map (Fig. 3a), the broad fringes (which curve upwards towards shorter wavelength with increasing angle of incidence) are due to Fabry-Perot interference in the underlying buffer layer and are not related to the operation of device. These are filtered from the data by applying a high pass spatial filter function to obtain Fig. 3b. The sharp narrow lines (dispersion bands) represent the coupling conditions for photonic crystal modes. The lower bands have linear behaviour and always show strongest coupling for photonic crystal modes. The fundamental band becomes nonlinear with respect to coupling angle around the Γ point (zero degree or normal incidence). The weaker bands with similar angular response indicate coupling to modes of the underlying SiO_2_ buffer layer. Examples are highlighted in Fig. 3. Because we focus on normal incidence at 785 nm, triangular lattice structure was investigated varying the lattice and hole diameter to calculate the precise parameters for both TE and TM modes. Figure 3c shows the resulting zero-order reflectance for each lattice type, the lower band corresponds to TE mode coupling and the higher band corresponds to TM mode coupling. The RCWA 12-fold lattice was omitted because it is incompatible with RCWA method. However, due to the similarity of six-fold lattice and the 12-fold lattice, the lattice constant and hole diameter will not have much change.

## Measurement and Discussion

### Angle-resolved dispersion map

**Figure 4:**
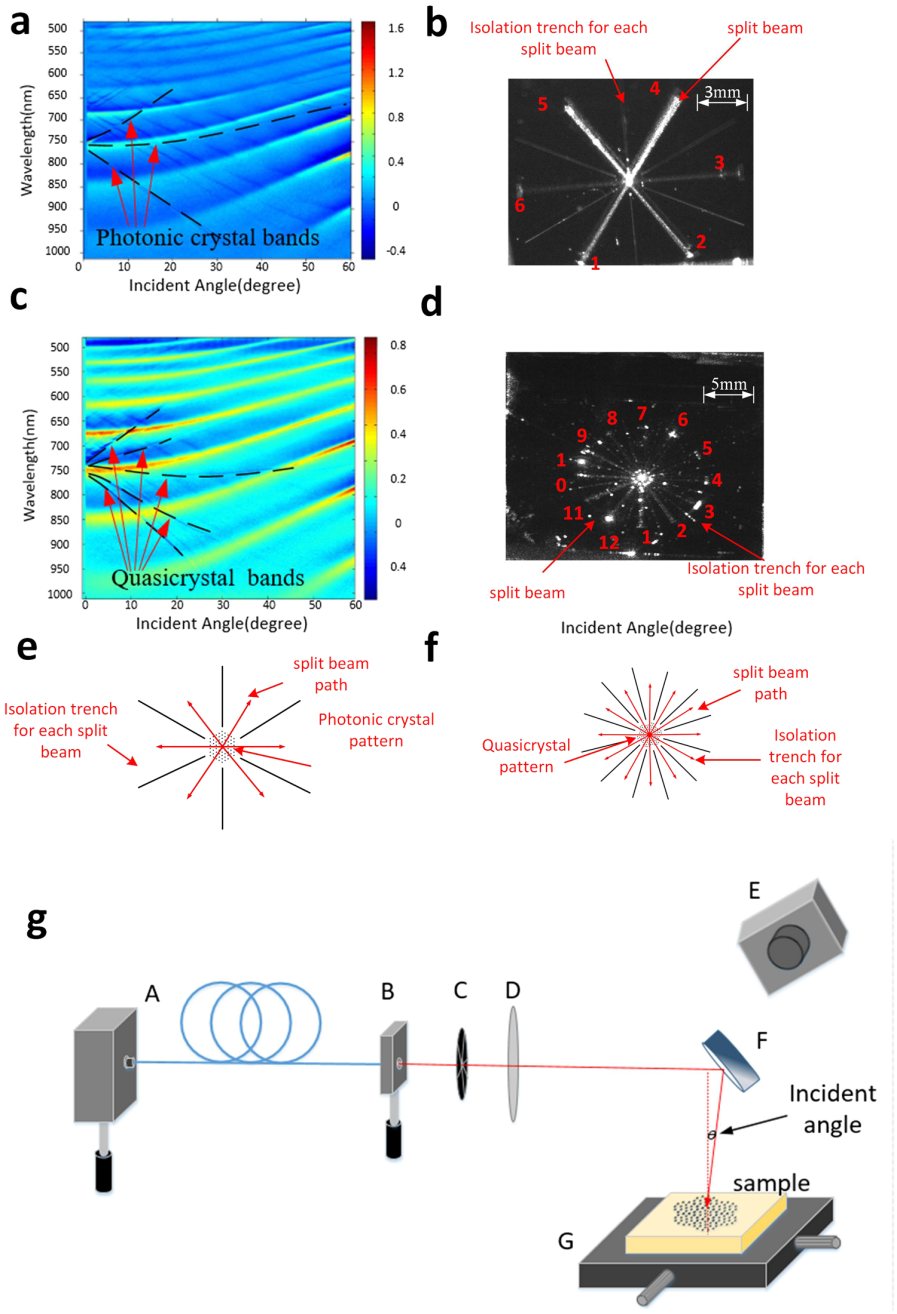
Performance measurement by reflectometry and visible coupling demonstration for devices on a silicon substrate. (**a**) Angle-resolved zero-order reflectance map of a triangular lattice coupler showing the coupling angle to be near 0 degree at 785 nm (raw experimental data). (**b**) Image of light coupling and beam splitting on a triangular coupler. (**c**) Angle-resolved zero-order reflectance map of quasi-crystal lattice coupler showing the coupling angle to be near 0 degree at 785 nm (raw experimental data). (**d**) Image of light coupling and beam splitting on a quasi-crystal lattice coupler. (**e**) Schematic of the triangular lattice coupler. (**f**) Schematic of the quasi-crystal lattice coupler (**g**) Schematic of the 785 nm coupling demonstration system.

The triangular and quasi-crystal couplers were characterized experimentally using both angle-resolved spectroscopic reflectometry and a bespoke setup designed to permit observation of the beam splitting. The detail of angle-resolved spectroscopic reflectometry is depicted in the methods section. Figure 4a and c shows angle-resolved spectroscopic reflectometry data for the triangular and quasi-crystal couplers. The photonic crystal mode lines (bands) precisely show the actual coupling conditions. Extremely good agreement with the simulation data is observed. The coupling angle is approximately 0 degree at 785 nm, and identical dispersion bands and Fabry-Perot interference fringes are observed in both simulation and experimental data. Normal incidence coupling matched theory extremely well for the triangular lattice, while there is about 20 nm offset for the 12-fold quasi-crystal coupler. The photonic crystal bands are indicated by the dashed black lines in Fig. 4(a and c). It is the fundamental bands (first band from high wavelength), which are used to diffract the beam in this design.

### Experimentally coupling at 785 nm

Near-vertical coupling and beam splitting on triangular and quasi-crystal devices with 785 nm light was successfully achieved, as shown in Fig. 4. The data in Fig. 4 comes from devices fabricated on silicon substrates and the images were collected using the configuration shown in Fig. 4g. The triangular coupler clearly shows 6-way splitting (Fig. 4b) while the quasi-crystal shows 12-way splitting (Fig. 4d), the individual beams are labelled with red numbers in Fig. 4b and d. Figure 4e and f show schematics of the triangular and quasi-crystal images shown in Fig. 4b and d respectively. For the quasi-crystal design the incidence angle is set to 4 degrees in order to achieve coupling. This small offset from vertical was due to variation in fabrication tolerance. Due to the offset, split beams have unequal intensities.

**Figure 5:**
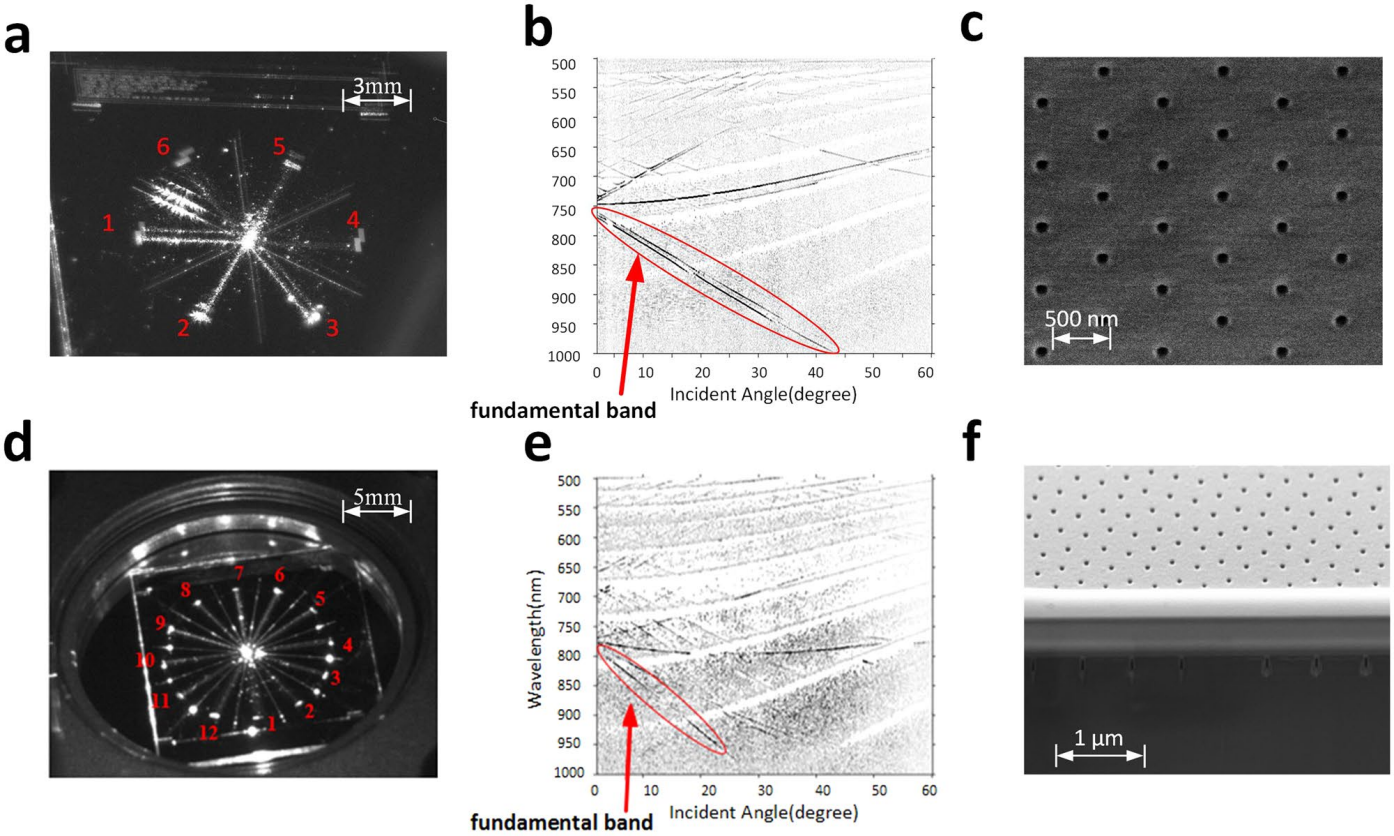
Light coupling on quasi-crystal structure with borosilicate glass substrate. (**a**) Image of 785 nm light coupling on a triangular lattice structure. (**b**) Angle-resolved zero order reflection measurement of a triangular lattice (filtered experimental data). (**c**) Top view SEM image of the triangular lattice structure. (**d**) Image of 785 nm light coupling on quasi-crystal structure. (**e**) Angle-resolved zero order reflection measurement on a quasi-crystal structure (filtered experimental data). (**f**) Cross section SEM image of the quasi-crystal structure.

The triangular and 12-fold quasi-crystal pattern was also fabricated on a borosilicate glass wafer. The incident coupling angle was 0° at 785 nm and equal intensity beam splitting with ‘perfect’ normal incidence angle was achieved. The filtered dispersion band diagrams and beam splitting images are shown in Figure. 5. Figure 5a and d show 785 nm light coupling to a triangular and quasicrystal lattice coupler respectively, using the same image collection as shown in Fig. 4g. Figure 5b and e show the zero-angle reflection experimental data for triangular and quasicrystal lattice couplers respectively and shows the photonic crystal dispersion bands with the fundamental band highlighted. Figure 5c shows a scanning electron microscope (SEM) of a triangular lattice device and Fig. 5f shows a cross-section SEM image of the quasi-crystal structure showing the depth and shape of the holes.

The reason why the small offset incidence angle causes uneven beam coupling, can be explained using the triangular lattice coupler (fabricated on the glass substrate) as an example. In Fig. 5a the coupling strength of the split beams 4, 5 and 6 are much weaker than that of beams 1, 2 and 3. This is because the small coupling angle causes a shift in centre position of the Ewald circle. Additional information relating to offset incidence angle on coupling behaviour is presented in the supplementary information.

## Conclusion

We have successfully demonstrated practical single photonic crystal/quasi-crystal devices, which serve the dual purpose of coupling incident light with large beam size to a planar slab waveguide and splitting the coupled light power to multiple guided beams simultaneously with benefit of small footprint. The experimental studies show very good agreement with theoretical predictions. The number of split beams depends on the symmetry of the photonic crystal lattice. By altering the lattice constant and hole diameters, phase matching can be achieved between the coupled light and photonic crystal lattice resulting in multiple beams. We also have shown that the number of coupled channels can be extended beyond the natural limit of six by using a quasi-crystal lattice. We have explained the theoretical design process in detail. Demonstrator devices were designed to couple and split ‘normally incident’ 785 nm light and were fabricated within a CMOS compatible SiON slab waveguide. In consequence this implementation of the photonic crystal coupler simplifies the monolithic integration of the traditional design and fabrication of grating coupler and power splitter. These photonic crystal structures can be applied to couple visible to infrared light without functional taper components. The scalable coupling area of the couplers is easy to match the size of source light from µm scale to mm scale and helps improve mode matching between the laser beam and the coupler. The large azimuthal angular coupling makes alignment easier than for conventional grating or photonic crystal couplers. They also provide confined and collimated split beams using a slab waveguide configuration instead of the conventional rib/ridge waveguide, providing high optical power throughput due to the highly multi-modal nature of the generated in-plane beams. This configuration also eliminates loss incurred by the rib/ridge waveguide fabrication process. The couplers are not limited to applications requiring high index contrast waveguides working at IR wavelength, but can be fabricated on low refractive index (SiON) material on transparent glass substrates enabling rear side light coupling. We propose that this combination of outstanding properties will be of great benefit to large area sensing applications, and provide an interesting and important new component for integrated optical devices.

## Methods

### FDTD modeling

The mode effective index of SiON is calculated by using commercial finite-difference time-domain software (Lumerical Solutions Inc.). A mode source with 785 nm was used in SiON/SiO_2_ structure. The fundamental TE_00_ and TM_00_ modes of slab waveguide were calculated, which gives the mode effective refractive index of 1.5881 and 1.5560, respectively.

### RCWA simulation

Rigorous Coupled Wave Analysis method are performed by commercial software (RSoft DiffractMOD) in order to calculate the Angle-resolved zero-order reflectance. A 3D model was established (see Figure S3). The incidence light had a wavelength from 500 nm to 900 nm with 1 nm resolution. The incidence angle was varied from 0° to 60° and the zero-order reflection was monitored.

### Device fabrication

The devices were fabricated on 150-mm silicon substrates using standard CMOS fabrication technology. A 2 μm thick SiO_2_ buffer layer, was grown by wet oxidation of the substrate at 1000 °C. A 400 nm thick SiON core layer was then deposited by plasma-enhanced chemical vapour deposition (PECVD, OPT PlasmaLab System 100). The targeted material refractive index of 1.7 was achieved by adjusting the gas flow ratio of SiH_4_, NH_3_, and N_2_. The ‘as grown’ optical loss of the SiON slab core layer (measured by prism coupling) was approximately 2 dB/cm. The wafer was annealed for 2 hours at 600 °C, reducing the slab waveguide loss to 0.2 dB/cm. Next, a 400 nm ZEP520 e-beam resist and charge dissipation layer (e-spacer) were spin coated onto the wafer. The photonic crystal patterns were defined by direct write electron beam lithography in the ZEP resist layer. Finally, the pattern was transferred into the SiON core by RIE etching (A cyclic etch process with CHF_3_ /Ar (25/25 sccm) and 200 W RF power). The residual resist was stripped by an overnight soak in NMP.

Double polished borosilicate glass substrates were used to fabricate a set of second generation devices. The borosilicate formed the underlying substrate used to support the slab waveguide core in replacement to silica-on-silicon substrate in the previous fabrication method. The most promising advantage provided by the transparent devices is the capability of rear side light coupling, which is useful for large area sensing applications. The fabrication process was similar to that developed for the silicon substrate devices. The only difference was the removal of the annealing process, which due to the low slumping temperature of the glass substrate, results in wafer bending.

Isolation trenches are used to physically separate each coupled beam on the slab waveguides. Each of the isolation trenches (shown in Fig. 4) were 12 µm wide lines etched into the SiON slab waveguide core, formed at the same time as the photonic crystal structure. These trenches are necessary to prevent crosstalk between adjacent paths. Due to weak reflections and scattering from the edges of the chip, these isolation trenches show up as bright lines on some of the images.

### Angle-resolved spectroscopic reflectometry

Angle-resolved spectroscopic reflectometry can be used to measure zero-order diffraction from the coupler over a broad spectral bandwidth^38^. A sophisticated reflectometry setup was used to collect the zero-order diffraction in order to determine the coupling angle of the coupler. It consists of a broadband laser light source, computer controlled motorized stages, a visible spectrometer and polarizer. The reflectometry system can collect zero-order reflectance data over an incident angle range of 0° to 90°. These measurements can be directly compared to the RCWA simulations.

### Light coupling setup

The setup (Fig. 4g) consists of a beam deflecting mirror (F), an X/Y stage (G) with a custom-made mount, a fixed-focus collimator (B) screwed into a rotation mount, an iris aperture (C), a 100 mm plano-convex lens (D), a 785 nm PM fibre pigtailed laser diode (A) with a typical output power of 7.5 mW, and a silicon CMOS camera to image the coupling (E). The 785 nm laser diode is the source of incident light and the fibre is terminated with the fixed-focus collimator in a rotation mount, which is used for selection of s/p polarization. S polarization is chosen in this case. The iris aperture is used to control the beam diameter and, by extension, the range of incident angles introduced by the plano-convex lens used to focus the beam on the surface. A larger beam diameter and angular range broadens the range of in-plane propagation angles for the coupled modes. Finally, the beam is reflected onto the stage-mounted sample by a rotating deflection mirror. The mirror and X/Y stage allow quick optimization of the input coupling conditions. The silicon CMOS cameras are used to capture the light coupling and splitting.﻿﻿﻿Data published in this paper are available from the University of Southampton repository at http://doi.org/10.5258/SOTON/D0059

## Electronic supplementary material

supplementary information
