# Supplementary material for: Photonic crystal and quasi-crystals providing simultaneous light coupling and beam splitting within a low refractive-index slab waveguide
Source: Sci Rep. 2017 May 12;7:1812. doi: 10.1038/s41598-017-01842-w (PMC5431799; doi:10.1038/s41598-017-01842-w)
Supplement: Supplementary file 1 — supplementary information [file 41598_2017_1842_MOESM1_ESM.pdf]

Supplementary information

Photonic crystal and quasi-crystals providing simultaneous light coupling and beam splitting within a low refractive-index slab waveguide.

JINGXING SHI[1,*], MICHAEL E. POLLARD[2], CESAR A. ANGELES[1], RUI QI CHEN[1], JAMES C. GATES[1] AND MARTIN D. B. CHARLTON[1]

[1]Faculty of Physical Sciences and Engineering, Building 53, University of Southampton, UK

[2]School of Photovoltaic and Renewable Energy Engineering, Tyree Energy Technologies Building, UNSW Australia, Sydney, Australia

**Corresponding author: sjxsoton@gmail.com*

Table of Contents

S1 Calculated diffraction patterns of a triangular lattice and quasicrystal lattice 2

S2 Comparison between near normal incidence and normal incidence coupling on a quasicrystal lattice 3

S3 3D Rsoft DiffractMOD model with different views 4

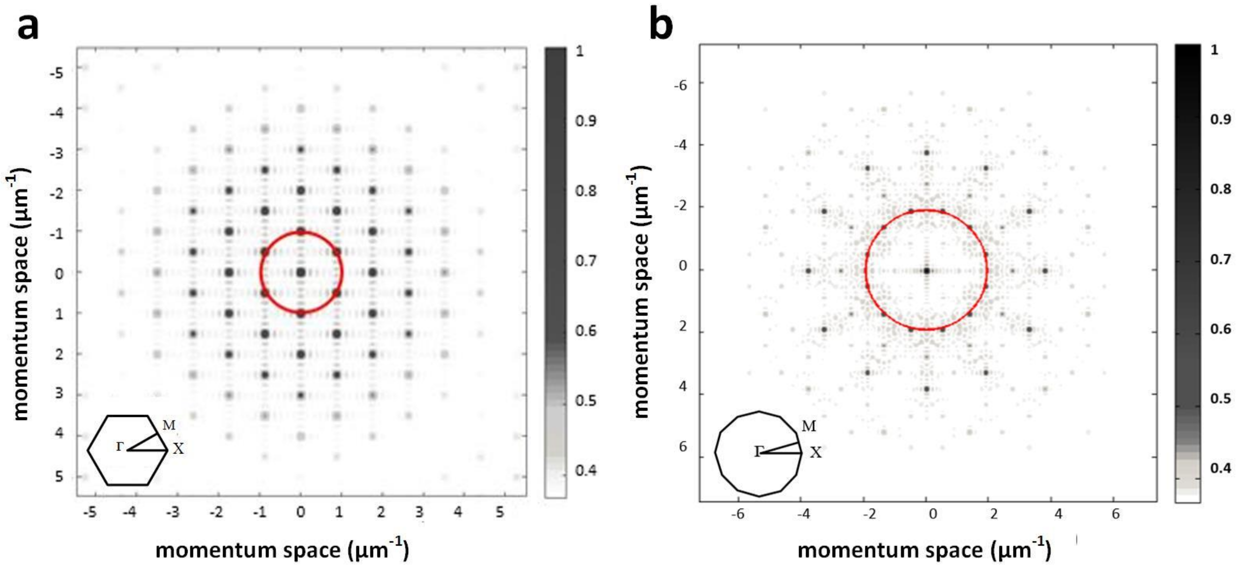

Figure S1. Calculated diffraction patterns of a triangular lattice and quasicrystal lattice. Red circles indicate Ewald construction for the first order Bragg peaks. a) hexagonal lattice. b) quasicrystal lattice

Diffraction pattern (reciprocal lattice) for the hexagonal photonic crystal and quasicrystal are shown in Figure S1. The diffraction pattern clearly demonstrates the expected 6-fold and 12-fold rotational symmetry for triangular lattice and quasicrystal lattice respectively, with the strongest points (Primary Bragg peaks) located in the inner circle (shown in the red circle). The Bragg peaks are calculated for the 1st Brillouin zone and the Γ point is shown as the insets in the figures.

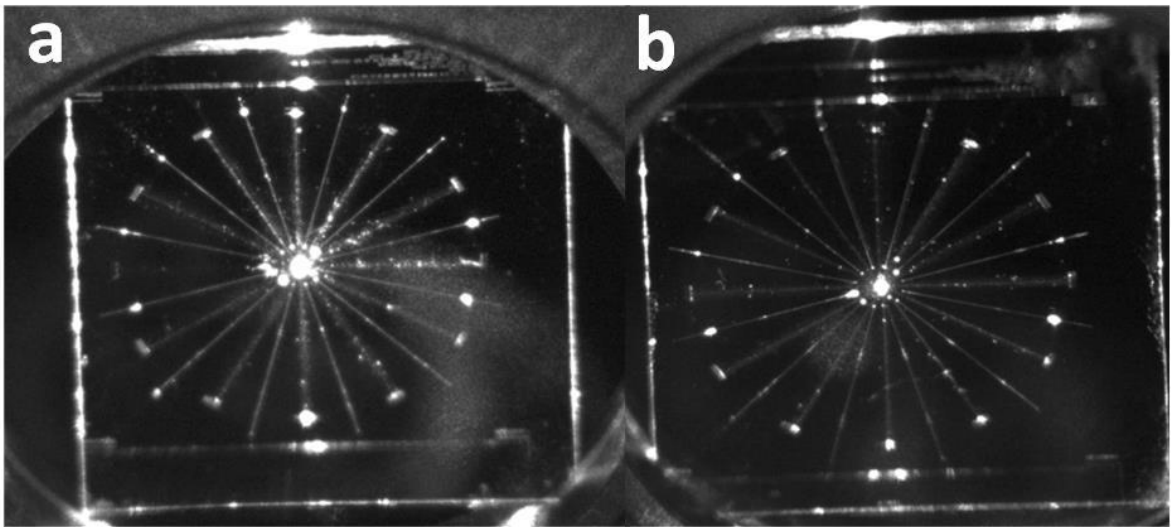

Figure S2. Comparison between near normal incidence and normal incidence coupling on a quasicrystal lattice. a) Near normal incidence coupling with an angle-offset of 0.2 degrees. b) Normal incidence coupling at 0 degrees.

Figure S2 shows a 12 fold quasi-crystal structure on a glass substrate with near normal incidence coupling (Fig. S2a) and normal incidence coupling (Fig. S2b). The comparison shows that stronger coupling is achieved with 'near-normal incidence' compared to 'normal incidence' alignment. However the greater overall increase in coupling is at the cost of un-even beam splitting, i.e. the coupled beams, on the left of Fig. S2a, is weaker than the coupled beams on the right of the figure. Figure S2b shows that more uniform beam coupling and splitting can be achieved with normal incidence alignment compared to Figure S2a.

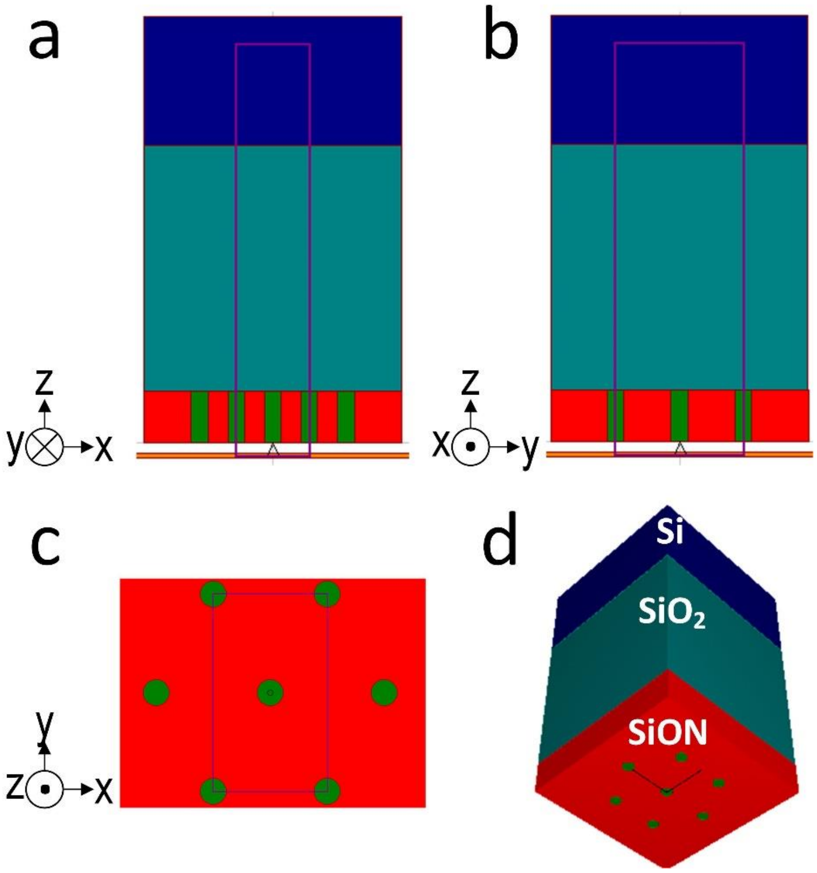

Figure S3. 3D Rsoft DiffractMOD model with different views. a) x-z plane, b) z-y plane, c) y-z plane and d) 3D structure. The green columns represent the air holes and dark purple solid lines define the simulation Boundaries.

Figure S3 shows the 3D Rsoft DiffractMOD model of a triangular lattice. The simulation window is depicted as purple lines and the boundary conditions are periodic along the x and y directions. The simulated incidence light wavelength was varied from 500nm to 900nm in 1nm increments. The incidence angle varied from 0˚ to 60˚ while the zero-order reflection was monitored. A key simulation parameter is the number of harmonics used to expand the refractive index and field in Fourier space. It was found that 5 harmonics achieved good refractive index resolution and resulted in reasonable simulation times.
